# Relationships Between Salivary Cortisol Slope and Neuropsychiatric Symptoms in Persons Living With Dementia

**DOI:** 10.1093/geroni/igab046.1693

**Published:** 2021-12-17

**Authors:** Yeji Hwang, Fanghong Dong, G Adriana Perez, Nancy Hodgson

**Affiliations:** 1 University of Pennsylvania, School of Nursing, Philadelphia, Pennsylvania, United States; 2 UPenn, Philadelphia, Pennsylvania, United States

## Abstract

While a flatter diurnal cortisol slope has been related to poor health outcomes in healthy populations, little is known about this relationship in persons living with dementia (PLWD). The purpose of this study was to examine the association between diurnalcortisol slope and neuropsychiatric symptoms in PLWD. Secondary data analysis was conducted using baseline data from the Healthy Patterns Study (N=168). Diurnal cortisol slope was calculated using the difference between changes in salivary cortisol from 30 minutes after awakening to bedtime. Spearman rho coefficients were used. Flatter cortisol slope was associated with the presence of symptoms of agitation (r=-0.191, p=0.013) and disinhibition (r=-0.168, p=0.03). Steeper cortisol slope was related to a more severe level of anxiety symptoms (r=0.36, p=0.009) and higher frequency of insomnia (r=0.292, p=0.011). We found that cortisol slope was associated with neuropsychiatric symptoms in PLWD. Future research is needed to examine the mechanisms underlying the relationships.

